# Use of primary and hospital care health services by chronic patients according to risk level by adjusted morbidity groups

**DOI:** 10.1186/s12913-021-07020-z

**Published:** 2021-10-03

**Authors:** Jaime Barrio-Cortes, María Soria-Ruiz-Ogarrio, María Martínez-Cuevas, Almudena Castaño-Reguillo, Mariana Bandeira-de Oliveira, María Teresa Beca-Martínez, María Carmen López-Rodríguez, María Ángeles Jaime-Sisó

**Affiliations:** 1 Primary Care Investigation Unit, Gerencia Asistencial de Atención Primaria, Madrid, Spain; 2 Foundation for Biosanitary Research and Innovation in Primary Care, Madrid, Spain; 3 Faculty of Health. Universidad Camilo José Cela, Madrid, Spain; 4 Healthcare Centre Ciudad Jardín, Gerencia Asistencial de Atención Primaria, Madrid, Spain; 5 Healthcare Centre Fuencarral, Gerencia Asistencial de Atención Primaria, Madrid, Spain; 6 Preventive Medicine Department, Hospital Virgen de la Salud. Complejo Hospitalario de Toledo, Toledo, Spain

**Keywords:** Chronic diseases, Multimorbidity, Risk levels, Morbidity classification, Health services, Primary care, Hospital care

## Abstract

**Background:**

Patients with chronic diseases have increased needs for assistance and care. The objective of this study was to describe the characteristics and use of primary care (PC) and hospital care (HC) health services by chronic patients according to risk level based on adjusted morbidity groups (AMG) and to analyze the associated factors.

**Methods:**

Cross-sectional descriptive observational study. Patients from a basic health area classified as chronically ill by the AMG classification system of the Madrid PC electronic medical record were included. Sociodemographic, clinical-care characteristics (classified as predisposing factors or need factors) and service utilization variables were collected. Univariate, bivariate and simple linear regression analyses were performed.

**Results:**

The sample consisted of 9866 chronic patients and 8332 (84.4%) used health services. Of these service users, 63% were women, mean age was 55.7 (SD = 20.8), 439 (5.3%) were high risk, 1746 (21.2%) were medium risk, and 6041(73.4%) were low risk. A total of 8226 (98.7%) were PC users, and 4284 (51.4%) were HC users. The average number of annual contacts with PC was 13.9 (SD = 15); the average number of contacts with HC was 4.8 (SD = 6.2). Predisposing factors associated with services utilization at both care levels were: age (B coefficient [BC] = 0.03 and 0.018, 95% CI = 0.017–0.052 and 0.008–0.028, respectively, for PC and HC) and Spanish origin (BC = 0.962 and 3.396, 95% CI = 0.198–1.726 and 2.722–4.070); need factors included: palliative care (BC = 10,492 and 5047; 95% CI = 6457–14,526 and 3098-6995), high risk (BC = 4631 and 2730, 95% CI = 3022–6241 and 1.949–3.512), number of chronic diseases (BC = 1.291 and 0.222, 95% CI = 1.068–1.51 and 0.103–0.341) and neoplasms (BC = 2.989 and 4.309, 95% CI = 1.659–4.319 and 3.629–4.989).

**Conclusions:**

The characteristics and PC and HC service utilization of chronic patients were different and varied according to their AMG risk level. There was greater use of PC services than HC services, although utilization of both levels of care was high. Service use was related to predisposing factors such as age and country of origin and, above all, to need factors such as immobility, high risk, and number and type of chronic diseases that require follow-up and palliative care.

**Supplementary Information:**

The online version contains supplementary material available at 10.1186/s12913-021-07020-z.

## Introduction

Patients with chronic diseases pose challenges for health systems. These pathologies have multiple impacts, are associated with high morbidity and mortality and are responsible for progressive deterioration, functional limitations, loss of autonomy, reduced quality of life and an increased use of health services, including both primary care (PC) [1, 2] and hospital care (HC) [3, 4].

In Spain, PC is the first level of care and the gateway to the health system. It is characterized by maximum accessibility and includes professionals in family medicine, pediatrics, nursing, dentistry, and social care work. PC offers comprehensive care that includes health promotion and education in healthy habits, prevention and monitoring of chronic diseases and orientation to social assistance programs. HC is the second level of care. It offers the most complex and costly diagnostic and/or therapeutic resources within the system (those whose efficiency is very low if they are not concentrated in one location), which are accessed by referral from a PC physician or in cases of urgency or vital risk that may require therapeutic measures that are only available in the hospital environment. HC fundamentally includes emergency room visits, outpatient visits, inpatient hospitalization, and day HC [5]. Coordination between the two levels of care has become a priority, especially for chronic patients, as a means of achieving continuity of care, reducing costs and improving the quality of care [4, 6, 7].

To respond to the needs of these patients, in recent years, multiple strategies to address chronicity have been implemented that seek to improve the care quality and improve the use efficiency of available resources. Many of these strategies are based on models that stratify the chronic population at different risk levels according to the Kaiser Permanente pyramid model (high-risk, medium-risk and low-risk chronic patients) [8, 9] or the King’s Fund, which includes health promotion and prevention for the general population together with health and social-care integrated vision [10]. For this stratification, morbidity classification systems are used. In Spain, one of the most frequently used classifiers in recent years has been adjusted morbidity groups (AMGs), which were developed with data from our health system and have been integrated into the electronic medical records (EMRs) of several PC autonomous communities [11]. These AMGs constitute a risk adjustment system that classifies individuals by taking their morbidity and complexity into account [8]. This new classification system is comparable to others that are available, such as adjusted clinical groups (ACGs) [12] and clinical risk groups (CRGs) [13], being proved more accurate than the latter for predicting PC visits, hospital admissions and pharmacy spending [14–16].

EMRs include data that allow an analysis of the relationship between the use of services and the factors that influence it, and they are accessible and reliable sources of information [17]. There are several models that provide a theoretical explanation of the health services utilization by system users and the influences of associated factors, but none of them are considered complete or definitive. Andersen [18] developed a behavioral model that explains how health services utilization is determined by a complex interaction of predisposing factors, need factors and facilitating factors. These factors are interrelated and favor or limit the level and frequency of service use [19].

Although there are studies on the usefulness of AMG and the stratification of chronic patients into different risk levels in the general population in PC [8, 20, 21], there is little information about the differences between PC and HC services utilization according to AMG risk levels.

The objective of this study was to describe the characteristics and use of PC and HC services of patients with chronic diseases patients according to their AMG risk level and to analyze the need and predisposing factors associated with this use.

## Materials and methods

### Study design, setting and sample

This was a cross-sectional descriptive study with an analytical approach. The study population comprised users from Ciudad Jardín healthcare centre, which has an enrolled population from 18,107. This centre is located in the Chamartín district in the northern area at the city of Madrid. It had a population of 143,424 with an average age of 45 years (23% >  65 years); 55% are women, 8.9% are foreigners, and the neighborhood has a low degree of socioeconomic deprivation [22, 23]. The study period was between June 2015 and June 2016. The study included patients who had a chronic disease according to the AMG stratification tool that has been integrated into the PC services of the community of Madrid. The chronic diseases considered by the classification tool are described in the Community of Madrid Strategy of Care for Patients with Chronic Diseases [24] and are reflected in Additional file 1: Appendix I. The AMG take into consideration several indicators to measure complexity based on morbidity (CBM): mortality, income (urgent, scheduled, medical and surgical), visits in primary care, external consultation, emergencies (primary care – specialized), prescription, outpatient hospital medication, day hospital, stays in social health centers, mental health. Through statistical modelling assign in each patient a numerical value of complexity (relative weight), which is the one that determines the risk level within assigning three cut-off points obtained from percentiles of the entire population [8, 25].

### Variables

The following variables for the use of PC services were collected: total annual contacts (a patient was considered a PC user if the number of annual contacts with PC was ≥1), type of contact (health, administrative or laboratory), form of contact (face-to-face, telephone, home visit) and professional contacted (family doctor, nurse, pediatrician, social worker, midwife, physiotherapist and dentist). For HC service use, the following variables were collected: total annual contacts (a patient was considered an HC user if the number of annual contacts with HC was ≥1), visits to outpatient clinics, emergency room visits, admissions, and day hospital visits.

Predisposing factors included gender, age and country of origin (Spain, Europe and the rest of the world). The need factors that were considered were being immobilized at home, being institutionalized, having a primary caregiver, receiving palliative care [26], number and type of chronic diseases, multimorbidity (≥ 2 chronic diseases), risk level (high, medium and low), AMG complexity index [8] and polymedication (patients with a medication regimen that implies having been prescribed five or more drugs for their chronic conditions). The low risk refers to patients with chronic conditions that are still in incipient stages. The medium risk refers to patients with chronic conditions that need disease-based approach. The high risk refers to highly complex patients, with multimorbidity and a multidisciplinary care approach.

This information was collected in PC EMRs and HC EMRs by the health professionals responsible for the patient care. The Madrid Information Health System Information Department extracted and anonymized the information in a database. The sociodemographic and clinical care data available were recorded as of June 30, 2015; for the PC and HC use variables, visits between June 30, 2015, and June 30, 2016, were considered.

### Statistical analysis

Qualitative variables are described as frequencies and percentages or as means with their corresponding standard deviation. The normality of the quantitative variables was determined. For comparisons of qualitative variables, the chi-square test was used, and the Mann-Whitney and Kruskal-Wallis U tests were used for quantitative variables. The Bonferroni method was used for multiple comparisons. The relationship between the number of annual contacts in PC and HC; and the predisposing and need factors of the Andersen model was analyzed using linear regression analysis, and an explanatory model was constructed that included factors that were statistically significant (*p* < 0.05) in the bivariate analysis. The final model was selected for its consistency with the theoretical model and according to the principle of parsimony; that is, between two possible similar models, the one that was simpler and required the fewest assumptions for its elaboration was chosen.

### Ethics approval

The study was approved by the Medical Research Ethics Committee of the La Princesa University Hospital and received a favorable report from the Local Research Commission of the Healthcare Directorate Center of Healthcare Management of the Community of Madrid.

## Results

A total of 9866 chronic patients were identified (54.4% of the total number of people assigned to the center), of whom 1534 (15.5% of all chronic patients) did not use PC or HC services and 8332 (84.5%) used these services. The patients who did not use PC or HC services had an average age of 46.7 years, compared to 57.4 years for those who used these services; 52.6% of nonusers were women, compared to 63% of users, and 70.9% of nonusers were of Spanish origin, compared to 83.9% of users. A total of 0.3 and 0.1% of patients who were not service users were immobilized and required primary caregivers, respectively, compared to 3.6 and 2.7% of users. Multimorbidity was present in 35.6% of non-service users, compared to 65.9% of users, and 1% of nonusers were polymedicated, compared to 19% of users. Among the nonusers, 0.2% were high-risk patients, compared to 5.3% of service users, while 97.8% of nonusers were low risk, compared to 73.3% of users. The predisposing and need factors of chronic patients according to their use of services are described in Table 1.

**Table 1:** Predisposing and need factors of chronic patients according to their use of services

Use of services n (%)	Total 9866 (100)	Nonuse of services 1534 (15.5)	Use of PC and/or HC services 8332 (84.5)	***p***
**Predisposing factors**				
Female gender	6056 (61.4)	807 (52.6)	5249 (63.0)	< 0.01
Age^a^	55.7 (20.8)	46.7 (16.4)	57.4 (21.1)	< 0.01
< 15 years	343 (3.5)	38 (2.5)	305 (3.7)	< 0.01
15–65 years	6040 (61.2)	1305 (85.1)	4735 (56.8)	
> 65 years	483 (35.3)	191 (12.5)	3292 (39.5)	
Origin Spain	8078 (81.9)	1087 (70.9)	6991 (83.9)	< 0.01
Europe	367 (3.7)	107 (7.0)	260 (3.1)	
Rest of the world	1421 (14.4)	340 (22.2)	1081 (13.0)	
**Need factors**				
Immobilized	300 (3.0)	4 (0.3)	296 (3.6)	< 0.01
Institutionalized	161 (1.6)	4 (0.3)	157 (1.9)	< 0.01
Primary Caregiver	229 (2.3)	1 (0.1)	228 (2.7)	< 0.01
Home support	80 (0.8)	1 (0.1)	79 (0.9)	< 0.01
Palliative care	44 (0.4)	2 (0.1)	42 (0.5)	0.04
Complexity weight^a^	6.7 (7.0)	2.8 (2.6)	7.4 (7.4)	< 0.01
High risk	444 (4.5%)	3 (0.2)	441 (5.3)	< 0.01
Medium risk	1784 (18.1%)	31 (2.0)	1753 (21)	
Low risk	7638 (77.4%)	1500 (97.8)	6138 (73.7)	
Multimorbidity	6036 (61.2)	546 (35.6)	5490 (65.9)	< 0.01
Chronic diseases^a^	2.5 (1.8)	1.6 (.9)	2.7 (1.9)	< 0.01
Anemia	908 (9.2)	136 (8.9)	772 (9.3)	0.6
Anxiety	2345 (23.8)	356 (23.2)	1989 (23.9)	0.6
Osteoarthritis	1055 (10.7)	50 (3.3)	1005 (12.1)	< 0.01
Asthma	1044 (10.6)	177 (11.5)	867 (14.4)	0.1
Ischemic heart disease	370 (3.8)	14 (0.9)	356 (4.3)	< 0.01
Cirrhosis	479 (4.9)	47 (3.1)	432 (5.2)	< 0.01
Dementia	213 (2.2)	5 (0.3)	208 (2.5)	< 0.01
Depression	1251 (12.7)	133 (8.7)	1118 (13.4)	< 0.01
Diabetes mellitus	1063 (10.8)	57 (3.7)	1006 (12.1)	< 0.01
Dyslipidemia	3780 (38.3)	379 (24.7)	3401 (40.8)	< 0.01
Dysrhythmias	696 (7.1)	40 (2.6)	656 (7.9)	< 0.01
OCPD	389 (3.9)	13 (0.8)	376 (4.5)	< 0.01
Glaucoma	395 (4.0)	18 (1.2)	377 (4.5)	< 0.01
Hypertension	3418 (34.6)	270 (17.6)	3148 (37.8)	< 0.01
Stroke	267 (2.7)	11 (0.7)	256 (3.1)	< 0.01
Heart failure	240 (2.4)	5 (0.3)	235 (2.8)	< 0.01
Chronic kidney disease	142 (1.4)	1 (0.1)	141 (1.7)	< 0.01
Active neoplasia	481 (4.9)	25 (1.6)	456 (5.5)	< 0.01
Obesity	1626 (16.5)	209 (13.6)	1417 (17)	< 0.01
Thyroid disorder	1646 (16.7)	171 (11.1)	1474 (17.7)	< 0.01
HIV	55 (0.6)	5 (0.3)	50 (0.6)	0.2
Polymedication	1598 (16.2)	16 (1.0)	1582 (19.0)	< 0.01

^a^Mean (standard deviation)

Of the total number of service users, 8226 (98.7%) used PC services, and 4284 (51.4%) used HC services. Among the PC users, 63.2% were women, the average age was 57.5 years, and 83% were Spanish; among the HC users, 61.3% were women, the average age was 60.4 years, and 93% were Spanish. A total of 3.6 and 2.8% were immobilized and had a primary caregiver compared in PC compared to 4.5 and 3.6% of those in HC. A total of 66.1 and 19.2% of PC users had multimorbidity and polymedication, respectively, compared to 73.4 and 25% of HC users, respectively. A total of 5.3% of PC users were classified as high risk, compared to 8.8% of HC users, while 62% of PC users were considered low risk, versus 73.4% of HC users. The predisposing and need factors of chronic patients according to their PC or HC service use are described in Table 2. These predisposing and need factors of chronic patients who used PC and HC services are stratified by sex and risk level in Tables 3 and 4, respectively.

**Table 2:** Predisposing and need factors of chronic patients according to their use of Primary Care (PC) or Hospital care (HC) services

Use of services n (%)	Use of PC services 8226 (98.7)	95% CI	Use of HC services 4284 (51.4)	95% CI
**Predisposing factors**				
Female gender	5201 (63.2)	62.2–64.3	2626 (61.3)	59.8–62.7
Age^a^	57.5 (21.1)	57.0–58.0	60.4 (20.4)	59.8–70.0
< 15 years	303 (3.7)	3.3–4.1	114 (2.7)	2.1–3.1
15–65 years	4643 (56.4)	55.4–57.5	2236 (52.2)	50.7–53.7
> 65 years	3280 (39.9)	38.2–40.9	1934 (45)	43.6–46.6
Origin Spain	6898 (83.9)	83.1–84.6	3,85 (93)	92.2–93.4
Europe	256 (3.1)	2.7–3.5	81 (1.9)	1.5–2.3
Rest of the world	1072 (13.0)	12.3–13.8	218 (5.1)	4.4–5.7
**Need factors**				
Immobilized	295 (3.6)	3.2–4.0	194 (4.5)	3.9–5.1
Institutionalized	157 (1.9)	1.6–2.2	88 (2.1)	1.6–2.5
Primary caregiver	227 (2.8)	2.4–3.1	154 (3.6)	3.0–4.1
Home support	78 (0.9)	0.7–1.1	50 (1.2)	0.8–1.5
Palliative care	42 (0.5)	0.3–0.7	35 (0.8)	0.5–1.1
Complexity weight^a^	7.5 (7.3)	7.3–7.6	9.4 (8.6)	9.1–9.6
High risk	439 (5.3)	4.8–5.8	376 (8.8)	7.9–9.6
Medium risk	1746 (21.2)	20.3–22.1	1251 (29.2)	27.8–30.6
Low risk	6041 (73.4)	72.2–74.4	2657 (62)	60.6–63.5
Multimorbidity	5541 (66.1)	65.1–67.2	3145 (73.4)	72.1–74.7
Chronic diseases^a^	2.7 (1.88)	2.6–2.8	3.1 (2.1)	3.0–3.2
Anemia	763 (9.3)	8.6–9.9	401 (9.4)	8.5–10.2
Anxiety	1962 (23.9)	22.9–24.8	1025 (23.9)	22.6–25.2
Osteoarthritis	996 (12.1)	11.4–12.8	638 (14.9)	13.8–15.9
Asthma	853 (10.4)	9.7–11.0	456 (10.6)	9.7–11.6
Ischemic heart disease	354 (4.3)	3.8–4.7	260 (6.1)	5.3–6.8
Cirrhosis	432 (5.3)	4.8–5.7	271 (6.3)	5.5–7
Dementia	206 (2.5)	2.2–2.8	127 (3)	2.4–3.5
Depression	1108 (13.5)	12.7–14.2	628 (14.7)	13.6–15.7
Diabetes mellitus	1004 (12.2)	11.5–12.9	630 (14.7)	13.6–15.8
Dyslipidemia	3377 (41.1)	40.0–42.1	1917 (44.7)	43.2–46.2
Dysrhythmias	651 (7.9)	7.3–8.5	449 (10.5)	9.6–11.4
COPD	374 (4.5)	4.1–5.0	275 (6.4)	5.7–7.1
Glaucoma	374 (4.5)	4.1–5.0	247 (5.8)	5.1–6.5
Hypertension	3129 (38)	3.0–39.1	1842 (43)	41.5–44.4
Stroke	255 (3.1)	2.7–3.5	177 (4.1)	3.5–4.7
Heart failure	234 (2.8)	2.5–3.2	183 (4.3)	3.7–4.9
Chronic kidney disease	141 (1.7)	1.4–2.0	116 (2.7)	2.2–3.2
Active neoplasia	451 (5.5)	5.0–6.0	341 (8)	7.1–8.8
Obesity	1408 (17.1)	16.3–18.0	813 (19)	17.8–20.0
Thyroid disorder	1464 (17.8)	17.0–18.6	812 (19)	17.8–20.0
HIV	46 (0.6)	0.5–0.7	40 (0.9)	0.6–1.2
Polymedication	1581 (19.2)	18.4–20.1	1073 (25)	23.7–26.3

^a^Mean (standard deviation)

**Table 3:** Predisposing and need factors of chronic patients using primary care (PC) by adjusted morbidity groups (AMG) risk levels and sex

PC users n (%)	High risk 439 (5.3)	Medium risk 1746 (21.2)	Low risk 6041 (73.4)	***P***	Female sex 5201 (63.2)	Male sex 3025 (36.8)	***p***
**Predisposing factors**							
Female gender	232 (52.8)	1131 (64.8)	3838 (63.5)	< 0.01	100	0	-
Age^a^	77.8 (12.8)	72.3 (14.9)	51.7 (20.1)	< 0.01	58.56 (20.9)	55.7 (21.3)	< 0.01
< 15 years	1 (0.2)	13 (0.7)	289 (4.8)		135 (2.6)	168 (5.6)	< 0.01
18–65 years	68 (15.5)	443 (25.4)	4132 (68.4)	< 0.01	2906 (55.9)	1737 (57.4)	
> 65 years	370 (84.3)	1290 (73.9)	1620 (26.8)		2160 (41.5)	1120 (37)	
Origin Spain	383 (87.2)	1542 (88.3)	4973 (82.3)		4363 (83.9)	2535 (83.8)	0.1
Europe	8 (1.8)	45 (2.6)	203 (3.4)	< 0.01	176 (3.4)	80 (2.6)	
Rest of the world	48 (10.9)	169 (9.1)	865 (14.3)		662 (12.7)	410 (13.6)	
**Need factors**							
Immobilized	123 (28)	125 (7.2)	47 (0.8)	< 0.01	222 (4.3)	73 (2.4)	< 0.01
Institutionalized	42 (9.6)	51 (2.9)	64 (1.1)	< 0.01	119 (2.3)	38 (1.3)	< 0.01
Primary caregiver	100 (22.8)	101 (5.8)	26 (0.4)	< 0.01	164 (3.2)	63 (2.1)	< 0.01
Home support	27 (6.2)	38 (2.2)	13 (0.2)	< 0.01	58 (1.1)	20 (0.7)	0.04
Palliative care	28 (6.4)	6 (0.3)	8 (0.1)	< 0.01	19 (0.4)	23 (0.8)	0.01
Complexity weight^a^	30.4 (12.5)	12.47 (2.7)	4.36 (2.2)	< 0.01	7. 3 (6.7)	7.7 (8.3)	0.02
Multimorbidity	436 (99.3)	1,687 (96.6)	3318 (54.9)	< 0.01	3508 (67.4)	1933 (63.9)	< 0.01
Chronic diseases^a^	6.7 (2.4)	4.3 (1.5)	1.9 (1.1)	< 0.01	2.8 (1.9)	2.6 (1.8)	< 0.01
Anemia	116 (26.4)	163 (9.3)	484 (8)	< 0.01	593 (11.4)	170 (5.6)	< 0.01
Anxiety	84 (19.1)	417 (23.9)	1461 (24.2)	< 0.01	1438 (27.6)	524 (17.3)	< 0.01
Osteoarthritis	108 (24.6)	424 (24.3)	464 (7.7)	< 0.01	771 (14.8)	225 (7.4)	< 0.01
Asthma	26 (5.9)	166 (9.5)	661 (10.9)	0,06	550 (10.6)	303 (10)	0.4
Ischemic heart disease	109 (24.8)	168 (9.6)	77 (1.3)	< 0.01	131 (2.5)	223 (7.4)	< 0.01
Cirrhosis	50 (11.4)	184 (10.5)	198 (3.3)	< 0.01	227 (4.4)	205 (6.8)	< 0.01
Dementia	55 (12.5)	90 (5.2)	61 (1)	< 0.01	160 (3.1)	46 (1.5)	< 0.01
Depression	101 (23)	382 (21.9)	625 (10.3)	< 0.01	862 (16.6)	246 (8.1)	< 0.01
Diabetes mellitus	187 (42.6)	425 (24.3)	2016 (33.4)	< 0.01	515 (9.9)	489 (16.2)	< 0.01
Dyslipidemia	296 (67.4)	1065 (61)	2016 (33.4)	< 0.01	2028 (39)	1349 (44.6)	< 0.01
Dysrhythmias	189 (43.1)	296 (17)	166 (2.7)	< 0.01	348 (7.4)	267 (8.8)	0.02
COPD	108 (24.6)	164 (9.4)	102 (1.7)	< 0.01	165 (3.2)	209 (6.9)	< 0.01
Glaucoma	44 (10)	155 (8.9)	175 (2.9)	< 0.01	125 (4.1)	249 (4.8)	0.2
Hypertension	362 (82.5)	1177 (67.4)	1590 (26.3)	< 0.01	1872 (36)	1257 (41.6)	< 0.01
Stroke	91 (20.7)	110 (6.3)	54 (0.9)	< 0.01	141 (2.7)	114 (3.8)	< 0.01
Heart failure	122 (27.8)	99 (5.7)	13 (0.2)	< 0.01	147 (2.8)	87 (2.9)	0.9
Chronic kidney disease	96 (21.9)	36 (2.1)	9 (0.1)	< 0.01	70 (1.3)	71 (2.3)	< 0.01
Active neoplasia	165 (37.6)	178 (10.2)	108 (1.8)	< 0.01	288 (4.4)	223 (7.4)	< 0.01
Obesity	131 (29.8)	455 (26.1)	822 (13.6)	< 0.01	861 (16.6)	547 (18.1)	0.08
Thyroid disorder	107 (24.4)	399 (22.9)	958 (15.9)	< 0.01	1227 (23.6)	237 (7.8)	< 0.01
HIV	5 (1.1)	11 (0.6)	30 (0.5)	0.3	7 (0.1)	39 (1.3)	< 0.01
Polymedication	348 (79.3)	767 (43.9)	466 (7.7)	< 0.01	1090 (21)	491 (16.2)	< 0.01

^a^Mean (standard deviation)

**Table 4:** Predisposing and need factors of chronic patients using hospital care by adjusted morbidity groups (AMG) risk levels and sex

Hospital care users n (%)	High risk 376 (8.8)	Medium risk 1251 (29.2)	Low risk 2657 (62)	***P***	Female sex 2626 (61.3)	Male sex 1658 (38.7)	***p***
**Predisposing factors**							
Female gender	198 (52.7)	794 (63.5)	1634 (61.5)		2626 (61.3)	0	
Age^a^	78 (12.2)	71.9 (14.7)	52.4 (19.5)	< 0.01	61.2 (20.4)	59,1 (20.4)	< 0.01
< 15 years	1 (0.3)	9 (0.7)	104 (3.9)		52 (2)	62 (3.7)	< 0.01
15–65 years	57 (15.2)	329 (26.3)	1850 (69.6)	< 0.01	1344 (51.2)	892 (53.8)	
> 65 years	318 (84.6)	913 (73)	703 (26.5)		1230 (46.8)	704 (42.5)	
Origin Spain	341 (90.1)	1181 (94.4)	2463(92.7)	0.032	2460 (93.7)	1525 (92)	0.01
Europe	6 (1.6)	23 (1.8)	52 (2)		53 (2)	28 (1.7)	
Rest of the world	29 (7.7)	47 (3.8)	142 (5.3)		113 (4.3)	105 (6.3)	
**Necessity factors**							
Immobilized	96 (25.5)	75 (6)	23 (0.9)	< 0.01	138 (5.3)	56 (3.4)	< 0.01
Institutionalized	31 (8.2)	27 (2.2)	30 (1.1)	< 0.01	65 (2.5)	23 (1.4)	0.014
Primary caregiver	80 (21.3)	61 (4.9)	13 (0.5)	< 0.01	106 (4)	48 (2.9)	0.051
Home support	22 (5.9)	24 (1.9)	4 (0.2)	< 0.01	31 (1.2)	19 (1.1)	0.92
Palliative care	25 (6.6)	5 (0.4)	5 (0.2)	< 0.01	16 (0.6)	19 (1.1)	0.057
Complexity weight^a^	30.6 (12.6)	12.7 (2.7)	4.8 (2.2)	< 0.01	9.2 (7.9)	9.7 (9.5)	0.41
Multimorbidity	372 (98.9)	1207 (96.5)	1566 (58.9)	< 0.01	1962 (74.7)	1183 (71.4)	0.015
Chronic diseases^a^	6.9 (2.4)	4.3 (1.5)	2.1 (1.2)	< 0.01	3.2 (2.1)	3.01 (2.02)	< 0.01
Anemia	99 (26.3)	115 (9.2)	187 (7)	< 0.01	292 (11.1)	109 (6.6)	< 0.01
Anxiety	75 (19.9)	289 (23.1)	661 (24.9)	0.08	732 (27.9)	293 (17.7)	< 0.01
Osteoarthritis	96 (25.5)	325 (26)	217 (8.2)	< 0.01	476 (18.1)	162 (9.8)	< 0.01
Asthma	25 (6.6)	120 (9.6)	311 (11.7)	< 0.01	315 (12)	141 (8.5)	< 0.01
Ischemic heart disease	92 (24.5)	124 (9.9)	44 (1.7)	< 0.01	93 (3.5)	167 (10.1)	< 0.01
Cirrhosis	44 (11.7)	133 (10.6)	94 (3.5)	< 0.01	139 (5.3)	132 (8)	< 0.01
Dementia	47 (12.5)	54 (4.3)	26 (1)	< 0.01	91 (3.5)	36 (2.2)	0.015
Depression	88 (23.4)	252 (20.1)	288 (10.8)	< 0.01	478 (18.2)	150 (9)	< 0.01
Diabetes mellitus	156 (41.5)	296 (23.7)	178 (6.7)	< 0.01	308 (11.7)	322 (19.4)	< 0.01
Dyslipidemia	255 (67.8)	758 (60.6)	904 (34)	< 0.01	1134 (43.2)	783 (47.2)	0.01
Dysrhythmias	166 (44.1)	211 (16.9)	72 (2.7)	< 0.01	257 (9.8)	192 (11.6)	0.062
COPD	95 (25.3)	124 (9.9)	56 (2.1)	< 0.01	114 (4.3)	161 (9.7)	< 0.01
Glaucoma	39 (10.4)	106 (8.5)	102 (3.8)	< 0.01	161 (6.1)	86 (5.2)	0.197
Hypertension	316 (84)	828 (66.2)	698 (26.3)	< 0.01	1084 (41.3)	758 (45.7)	< 0.01
Stroke	80 (21.3)	69 (5.5)	28 (1.1)	< 0.01	98 (3.7)	79 (4.8)	0.098
Heart failure	110 (29.3)	67 (5.4)	6 (0.2)	< 0.01	117 (4.5)	66 (4)	0.45
Chronic kidney disease	84 (22.3)	26 (2.1)	6 (0.2)	< 0.01	58 (2.2)	58 (3.5)	0.011
Active neoplasia	144 (38.3)	137 (11)	60 (2.3)	< 0.01	156 (5.9)	185 (11.2)	< 0.01
Obesity	112 (29.8)	322 (25.7)	379 (14.3)	< 0.01	495 (18.8)	318 (19.2)	0.789
Thyroid disorder	91 (24.2)	279 (22.3)	442 (16.6)	< 0.01	671 (25.6)	141 (8.5)	< 0.01
HIV	5 (1.3)	7 (0.6)	28 (1.1)	0.22	4 (0.2)	36 (2.2)	< 0.01
Polymedication	307 (81.6)	541 (43.2)	225 (8.5)	< 0.01	708 (27)	365 (22)	< 0.01

^a^Mean (standard deviation)

Among the PC users, the average number of annual contacts was 13.9. The average number of healthcare contacts was 11.9. The average number of face-to-face contacts was 12.7. Family doctors were contacted for an average of 7.2 visits, nurses had an average of 3.8 contacts, pediatricians had an average of 0.3, physical therapists had an average of 0.3, and social workers had an average of 0.08. Of the patients who used HC, the average number of annual contacts was 4.8. The average number of visits to outpatient clinics was 3.7, followed by an average of 0.7 visits to the emergency room, 0.3 visits to the day hospital and 0.2 hospitalizations. The use of PC and HC services stratified according to risk level, gender and age groups is shown in Table 5.

**Table 5:** Annual use of primary care and hospital care services by chronic patients according to adjusted morbidity groups (AMG) risk level, sex and age group

**Primary care users** Mean (SD)	**Total** 8226 (100%)	**95% CI**	**High risk** 376 (8.8%)	**Medium risk** 1251 (29.2%)	**Low risk** 2657 (62%)	***p***	**Female sex** 2626 (61.3%)	**Male sex** 1658 (38.7%)	***p***	**< 15** **years** 303 (3.7)	**15–65** **years** 4643 (56.4)	**> 65** **years** 3280 (39.9)	***p***
***Annual total***	13.9 (15)	13.5–14.2	34.3 (27.9)	21.9 (17.2)	10.1 (10.1)	< 0.01	14.3 (14.4)	13.1 (16)	< 0.01	9.8 (8.3)	9.9 (11)	19.5 (18.2)	< 0.01
***Contact type***													
Health contact	11.9 (13.1)	11.6–12.2	29.2 (25.8)	18.8 (14.9)	8.6 (8.7)	< 0.01	12.3 (12.5)	11.2 (4.1)	< 0.01	9.3 (7.9)	8.5 (9.2)	16.9 (16.2)	< 0.01
Administrative contact	1.0 (3.5)	0.9–1.1	3.3 (6.8)	1.9 (4.9)	0.6 (2.5)	< 0.01	1.01(3.4)	1.1 (3.9)	< 0.01	0.2 (1)	0.7 (1.1)	1.1 (1.6)	< 0.01
Laboratory contact	0.9 (1.4)	0.8–0.9	1.8 (2.4)	1.2 (1.5)	0.7 (1.5)	< 0.01	0.9 (1.4)	0.8 (1.3)	< 0.01	0.2 (0.6)	0.7 (1.1)	1.1 (1.6)	< 0.01
***Form of contact***													
In-person	12.7 (12.7)	12.4–13.0	26.7 (22)	19.5 (14.8)	9.7 (9.5)	< 0.01	13 (12.2)	12.2 (13.6)	< 0.01	9.6 (7.9)	9.8 (10.5)	17.1 (14.6)	< 0.01
Telephone	0.4 (2.4)	0.3–0.5	2.4 (7.9)	0.91 (2.9)	0.2 (0.8)	< 0.01	0.5 (2.7)	0.4 (1.9)	< 0.01	0.2 (0.9)	0.2 (1.2)	0.8 (3.5)	< 0.01
Home visit	0.7 (4.1)	0.6–0.8	5.1 (12.3)	1.5 (5)	0.2 (1.7)	< 0.01	0.8 (3.7)	0.6 (4.7)	< 0.01	0 (0.1)	0.1 (0.9)	1.7 (6.2)	< 0.01
***Professional contacted***													
Physician	7.2 (7.1)	7.1–7.4	15.9 (11.9)	11.1 (8.2)	5.5 (5.2)	< 0.01	7.7 (7.4)	6.5 (6.7)	< 0.01	0.2 (0.6)	5.9 (5.9)	9.7 (8.1)	< 0.01
Pediatrician	0.3 (1.8)	0.2–0.3	0.03 (0.6)	0.2 (2)	0.3 (1.7)	< 0.01	0.2 (1.7)	0.3 (1.8)	< 0.01	6.4 (6)	0.03(0.9)	0.01 (0.3)	< 0.01
Nurse	3.8 (7.5)	3.6–3.9	12.9 (18.6)	6.6 (8.4)	2.3 (4.6)	< 0.01	3.7 (6.3)	3.9 (9.3)	0.07	0.03 (0.5)	0.3 (1.9)	0.5 (2.5)	< 0.01
Physical therapist	0.3 (2.1)	0.2–0.4	0 (0)	0.6 (2.8)	0.3 (2)	< 0.01	0.4 (2.3)	0.2 (1.8)	0.01	0 (0.1)	0.2 (1)	0.01 (0.1)	< 0.01
Midwife	0.1 (0.8)	0.09–0.1	0.01 (0.1)	0.05 (0.4)	0.2 (0.9)	< 0.01	0.2 (1)	0 (0.1)	< 0.01	0.4 (0.9)	0.1 (0.5)	0.03 (0.3)	< 0.01
Dentist	0.07 (0.4)	0.06–0.08	0 (0)	0.1 (0.5)	0.1 (0.4)	< 0.01	0.1 (0.4)	0.1 (0.5)	0.2	0 (0)	0.03 (0.3)	0.2 (1)	< 0.01
Social worker	0.08 (0.7)	0.07–1.0	0.3 (1.5)	0.2 (1)	0.04 (0.4)	< 0.01	0.1 (0.7)	0.8 (0.7)	0.3	0 (0)	0.03 (0.3)	0.2 (1)	< 0.01
**Hospital care users** Mean (SD)	**Total** 4284 (100%)	**95% CI**	**High risk** 376 (8.8%)	**Medium risk** 1251 (29.2%)	**Low risk** 2657 (62%)	***p***	**Female gender** 2626 (61.3%)	**Male gender** 1658 (38.7%)		**< 15** **Years** 114 (2.7%)	**15–65** **Years** 2236 (52.2%)	**> 65** **Years** 1934 (45%)	***p***
***Annual total***	4.8 (6.2)	4.6–5.0	10 (11.9)	5.9 (5.9)	3.5 (4.8)	< 0.01	4.6 (5)	5.2 (7.7)	< 0.01	0.7 (1.2)	2 (4.4)	5.7 (6.8)	< 0.01
***Contact type***													
External consultations	3.7 (4.2)	3.6–3.8	6.6 (6.7)	4.6 (4.7)	2.8 (3)	< 0.01	3.6 (3.9)	3.9 (4.8)	< 0.01	1.1 (1.3)	3.3 (3.9)	4.3 (4.6)	< 0.01
Emergencies	0.7 (1.2)	0.6–0.8	1.4 (1.8)	0.8 (1.4)	0.5 (0.9)	< 0.01	0.7 (1.2)	0.7 (1.2)	0.6	0.5 (0.8)	0.6 (1)	0.8 (1.4)	< 0.01
Income	0.2 (0.4)	0.1–0.3	0.5 (0.9)	0.2 (0.5)	0.1 (0.3)	< 0.01	0.1 (0.4)	0.2 (0.5)	0.7	0.2 (0.5)	0.1 (0.3)	0.2 (0.6)	< 0.01
Day hospital care	0.3 (2.8)	0.2–0.4	1.4 (5.7)	0.2 (1.5)	0.1 (2.5)	< 0.01	0.2 (1.5)	0.4 (4)	0.2	0.1 (0.4)	0.3 (3)	0.3 (2.5)	< 0.01

After multivariate adjustment according to behavioral model factors, female gender was associated with greater use of PC services (B coefficient (BC) = 0.675, 95% CI = 0.082; 1.268), age (BC = 0.035, 95% CI = 0.017; 0.052), as was Spanish origin (BC = 0.962, 95% CI = 0.198; 1.726), immobilization (BC = 8.129; 95% CI = 6.437; 9.822), the need for palliative care (BC = 10.492; 95% CI = 6.457; 14.526); high risk level (BC = 4.631; 95% CI = 3.022; 6.241), number of chronic diseases (BC = 1.291; 95% CI = 1.068; 1.510), diabetes mellitus (BC = 2.332, 95% CI = 1.375; 3.290), heart failure (BC = 3243; 95% CI = 1361; 5125), stroke (BC = 3415, 95% CI = 1707; 5122), dementia (BC = 5267; 95% CI = 1376; .157), neoplasia (BC = 2,89); 95% CI = 1.659; 4.319) and polymedication (BC = 6.600; 95% CI = 5.649; 7.552). Age (BC = 0.018, 95% CI = 0.017; 0.052), Spanish origin (BC = 3.396, 95% CI = 2.722; 4.070), and immobilization (BC = − 1.522; 95% CI = − 2.422; − 0) were associated with the HC model, 62), as were the need for palliative care (BC = 5.047; 95% CI = 3.098; 6.995), high risk level (BC = 2.730; 95% CI = 1.949; 3.512), number of chronic diseases (BC = 0.222; 95% CI = 0.103; 0.341), COPD (BC = 1.349 95% CI = 0.616; 2.082), depression (BC = 0.723; 95% CI = 0.221; 1.220), dementia (BC = − 1.504; 95% CI = -2.560; − 0.448) and active neoplasia (BC = 4.309; 95% CI = 3.629; 4.989) (Table 6).

**Table 6:** Factors associated with chronic patients’ use of primary care and hospital care

Predisposing and need factors	B coefficient (95% CI)	***p***
**Primary care users**		
Female gender	0.675 (0.082; 1.268)	0.000
Age^a^	0.035 (0.017;0.052)	0.026
Spanish origin	0.962 (0.198; 1.726)	0.014
Immobilized	8.129 (6.437; 9.822)	0.014
Palliative care	10.492 (6.457; 14.526)	0.000
High risk level	4.631 (3.022; 6.241)	0.000
Number of chronic diseases^a^	1.291 (1.068; 1.51)	0.000
Diabetes mellitus	2.332 (1.375; 3.290)	0.000
Heart failure	3.243 (1.361; 5.125)	0.000
Stroke	3.415 (1.707; 5.122)	0.000
Dementia	5.267 (3.376; 7.157)	0.000
Active neoplasia	2.989 (1.659; 4.319)	0.000
Polymedication	6.600 (5.649; 7.552)	0.000
R2 = 0.254		
**Hospital care users**		
Age^a^	0.018 (0.008; 0.028)	0.001
Spanish origin	3.396 (2.722; 4.070)	0.000
Immobilized	−1.522(−2.422; −0.62)	0.001
Palliative care	5.047 (3.098; 6.995)	0.000
High risk level	2.730 (1.949; 3.512)	0.000
Number of chronic diseases^a^	0.222(0.103; 0.341)	0.000
Active neoplasia	4.309 (3.629; 4.989)	0.000
COPD	1.349 (0.616; 2.082)	0.000
Depression	0.723 (0.221; 1.22)	0.005
Dementia	−1.504 (−2.560; −0.448)	0.005
R2 = 0.151		

^a^Quantitative variables

## Discussion

A large majority of the chronic patients were health service users. These service users were older, with a predominance of female gender and Spanish nationality, greater morbidity, increased presence of serious diseases and a greater need for assistance, care and medication than those who did not use services. The use of both PC and HC services was high, with greater use of PC than HC, and an increase in the use of both levels of care with increasing risk level and age. The factors associated with the use of both PC and HC included predisposing factors, such as age and Spanish origin, and need-related factors, such as the need for palliative care, high risk, number of chronic diseases and neoplasms.

### Characteristics of the patient population and the use of PC and HC services

The chronic patients had a high average age and a predominance of Spanish nationality. Almost two-thirds were women and had multimorbidity (the most frequent of which chronic cardiovascular diseases, osteoarticular diseases and neoplasms), and more than a quarter were polymedicated. These data can be considered representative of the Community of Madrid, and their distribution is correlated with the total stratification pyramid published by the Community [11] and is similar to others European studies with similar objectives [27–29].

A large majority of chronic patients used PC services, and patients at all risk levels had a high average number of annual contacts with PC. The majority of the contacts were health contacts and took place in person, as observed in other studies [30–32]. The low number of non-face-to-face contacts and home visits stood out; this is a potential area of care for chronic patients that should be strengthened, particularly in light of the COVID-19 pandemic. The average number of contacts with family doctors in our study was lower than the average number of physician consultations reported in comparable studies [33, 34]. However, it was almost double number of contacts with nurses, unlike previous studies in which the average number of contacts with nurses was higher than with doctors [35]. This lower number of nursing contacts in our study is striking and should prompt a reflection on the care provided for these patients, since strategies for addressing chronicity which postulate models of care that enhance and prioritize care and follow-up by the PC nursing staff and reserve contact with the PC family physician for issues that require medical attention [24]. Along this line, despite the good socioeconomic indicators of the health service area in general, it would be expected that some chronic patients would have increased needs for social assistance; nonetheless, a very low number of contacts with the social worker was observed. The coverage of several healthcare centers by a single social worker could lead to low accessibility and raises the need to strengthen this ratio to offer comprehensive social health care. Among the PC users, all types and forms of contact and all professional contact increased significantly according to the level of risk; the exception was for physical therapists, midwives and dentists, which could be explained because these professionals usually attend to patients who are younger and have lower risk and lower morbidity. In other studies of patients with similar characteristics to those of our high-risk population, such as complex chronic or multiple pathologies, a high degree of contact with PC professionals is also observed in both developed and developing countries [32, 36–38]. The average number of annual contacts with PC was higher in women than in men, regardless of their risk level, and women had significantly more health contacts, home visits and contacts with doctors than men did. These results are similar to those of other studies [36, 37]. Among the possible explanations for this increased utilization are that women report a worse perceived state of health and have a higher prevalence of minor affective disorders that can generate the need for health consultations; additionally, women are also often caregivers or heads of the household, which could favor a need to consult health services on behalf of other family members [34]. In contrast, nurse consultations were more frequent among high-risk men, which could be due to less self-care capacity among men, especially those in older age ranges [34]. The results show that increased age was associated with a major number of contacts for most types and forms and with professionals other than pediatricians, midwives, physical therapists and dentists. This is because age is one of the most influential modifiers of utilization, as observed in many series [38–40].

The average use of HC services by these chronic patients was also very high. The majority of contacts were outpatient consultations, followed by emergencies, day hospital care and hospitalizations. The total number of contacts with HC for all types of visits increased significantly according to risk level. There is no literature that compares the use of hospital services by patients stratified according to AMG, but these data correlate with what is described in the literature for patients with frailty, functional deterioration or high-complexity needs [41, 42], who are similar to high-risk patients; in patients with multimorbidity or multiple pathologies [43, 44], who are similar to medium-risk patients; and in patients with a single chronic disease [1], who are similar to low-risk patients. Regarding sex, the average number of annual contacts with HC, visits to outpatient clinics and admissions was higher in men, which is in line with the results of studies that show that women are hospitalized significantly less often than men [45]. Regarding age and the use of HC services, chronic patients under 15 years of age had a lower average use of services, while use increased among patients aged 15 to 65 years and was the highest among those older than 65 years, as observed in other studies [42, 44, 46]. This high use of PC and general HC services can be favored by inadequate management of resources and a lack of care continuity, as has been identified by studies in our environment [6, 47]. To improve continuity of care, transformative and innovative leadership by organizations is necessary, as seen in evidence from the United States, Europe and Spain from new care models for chronic patients that have increased the quality, efficiency and sustainability of health systems, improved health outcomes and reduced the number of emergency room visits, admissions and readmissions [48].

### Factors associated with the utilization of services

According to the literature, the use of health services depends mainly on the user’s predisposing and need factors [49]. Based on the Andersen model, the predisposing factors that determined the use of both PC and HC services were age, in line with the existing literature [38–40, 42, 44, 46], and Spanish origin, which may be because people of other nationalities may have less access to health care and the enactment of Royal Decree Law 16/2012 in 2012, where it is described that foreign people in an irregular situation lost the right of access to standardized health care. The need factors that influenced the use of services at both levels of care were the need for palliative care, high risk, the number of chronic diseases and neoplasms.

For PC users, predisposing factors such as female gender were specifically associated with greater use of services, as is also reflected in the literature [36, 37]; to a greater extent, need factors such as being immobilized and presenting diseases that require polymedication and frequent monitoring or palliative care (diabetes mellitus, heart failure, stroke, dementia) influenced PC use, consistent with other studies [38, 39, 46].

Among HC users, there was a lower use among immobilized patients and patients with dementia, which is explained because care for this group primarily entails home visits from PC services. In contrast, among HC users, greater use by patients with other diseases, such as depression, stood out, which coincides with the literature and is explained by the coexistence of these diseases with a variety of other chronic diseases that require hospital care, such as heart diseases, cerebrovascular accidents, cancer or diabetes [47] and COPD, which is one of the main reasons for visits to outpatient clinics and emergency rooms and hospital admissions [48, 49].

The risk levels are intended to guide PC and HC health professionals through assigning a specific intervention and coordinated level of care to each chronic patient, along with their knowledge of that patient and its context. This offers a more individualized and patient-centered attention based on the Kaiser Permanente stratification model. The goal for low risk patients is to slow the progression of the disease and prevent the patient from reaching higher levels of risk. To this end, the self-management of the disease and the education of a preventive nature and healthy habits are supported to avoid healthcare utilization. The goal for patients at medium risk is to slow progression by planning and managing the disease that combines self-management and professional care. The high risk patients have an increased utilization of healthcare services and the objective at this level is to reduce flare-ups and hospital admissions through comprehensive case management, with mainly professional care [24]. This ideal service selection correlates with current service utilization observed in the study with an increased healthcare services utilization in the high-risk level both in PC and HC.

### Strengths and limitations

As a cross-sectional study, the nature of the associations cannot be interpreted in terms of cause-effect. On the other hand, the existence of information biases due to mistakes or errors in diagnostic coding could limit the ability of the EMR to reflect real morbidity, but most ICPC-2- diagnostic codes and the rest of clinical and contacts information are recorded by the health professional responsible for the patient care. Additionally, the use of secondary clinical-administrative sources makes it possible to work with almost all individuals and not with partial samples or volunteers, which minimizes possible selection and memory biases. The existence of patients who may not be represented in the total population of the center because they were not contacted or did not use PC services due to having double insurance is unlikely to significantly alter the results since the proportion of people with a health care card in Madrid reaches 95% [17]. In our study, there were no factors related to the service provider or organization that were considered likely to influence the use of the service, since those data are not available. However, most use of services is usually attributed to factors related to the user [49]. Regarding AMGs, doubts have been raised about their transparency, although this is a situation common to commercial classification tools, as stated by Huntley et al. [2]. As a result of this, it’s not exactly known how specifically each different factor is weighted in the complexity index per CBM. Besides, AMGs have a clinical-care management purpose that considers the complexity and morbidity of the patient but does not take into account other factors, such as their psychosocial and socioeconomic situation. Despite this, there has been strong agreement among classifiers, and AMGs have shown good predictive capacity [14]. The developers of AMGs have shown that they are a useful tool that allows the detection of comparable health centers and facilitates the study of variability in the consumption of resources and other clinical-organizational aspects [8]. Therefore, the Ministry of Health, Social Services and Equality is integrating them into the EMR of the National Health System in autonomous communities for the management of chronic patients [8, 20].

## Conclusions

The characteristics and use of PC and HC services by chronic patients differed and varied according to the patients risk level, as determined by their AMG. PC service use was higher than HC use, although the use of both levels of care was high. The most frequent type of contact in PC was with a health care provider, specifically a family doctor, while in HC, it was outpatient consultation and emergency care. The use corresponded with predisposing factors such as age and country of origin and, above all, with need factors such as immobility, high risk, the number and type of chronic diseases and the need for monitoring and palliative care.

## Supplementary Information

**Additional file 1.**

## Data Availability

The datasets generated and analysed during the current study are not publicly available due the belong to the Madrid Health Service electronic medical record but are available from the corresponding author on reasonable request.
