# Supplementary material for: Use of primary and hospital care health services by chronic patients according to risk level by adjusted morbidity groups
Source: BMC Health Serv Res. 2021 Oct 3;21:1046. doi: 10.1186/s12913-021-07020-z (PMC8487403; doi:10.1186/s12913-021-07020-z)
Supplement: Supplementary file 1 — Additional file 1. [file 12913_2021_7020_MOESM1_ESM.docx]

**TITLE:** Use of primary and hospital care health services by chronic patients according to risk level by adjusted morbidity groups.

**AUTHORS:**

Jaime Barrio-Cortes (1, 2, 3) *.

María Soria-Ruiz-Ogarrio(4).

María Martínez-Cuevas (5).

Almudena Castaño-Reguillo (4).

Mariana Bandeira-de Oliveira (4).

María Teresa Beca-Martínez (6).

María Carmen López-Rodríguez (4).

María Ángeles Jaime-Sisó (4).

(1) Primary Care Investigation Unit. Gerencia Asistencial de Atención Primaria. Madrid. Spain.

(2) Foundation for Biosanitary Research and Innovation in Primary Care. Madrid. Spain.

(3) Faculty of Health. Universidad Camilo José Cela. Madrid. España.

(4) Healthcare Centre Ciudad Jardín. Gerencia Asistencial de Atención Primaria. Madrid. Spain.

(5) Healthcare Centre Fuencarral. Gerencia Asistencial de Atención Primaria. Madrid. España.

(6) Preventive Medicine Department. Hospital Virgen de la Salud. Complejo Hospitalario de Toledo. Spain.

***Corresponding Author:** Jaime Barrio Cortes. MD, PhD. Primary Care Investigation Unit. Calle San Martín de Porres, 6. 5th floor. 28035. Madrid, Spain.

Email: jaime.barrio@salud.madrid.org. Phone: (+34) 660117699.

**Appendix 1. Types of chronic diseases considered by the Adjusted Morbidity Group (AMG) in the Community of Madrid at the time of data extraction**

| Medical Diagnosis | ICP-2 codes |
| --- | --- |
| Alcoholism | P19 |
| Anaemia | B78-82 |
| Aorta aneurysm | K92 |
| Anxiety | P82 |
| Arthritis | L88 |
| Asthma | R96 |
| Attention-Deficit/Hyperactivity Disorder | P71-72 |
| Bladder cancer | U76 |
| Breast cancer | X76 |
| Cardiopulmonary disease | K82 |
| Central nervous system cancer | N74 |
| Cervical cancer | X75 |
| Cirrhosis | D97 |
| Colon cancer | D75 |
| Dementia | P78 |
| Depression | P86 |
| Diabetes Mellitus | T89-90 |
| Dyslipidaemia | T93 |
| Dysrhythmias | K78-80 |
| Ear, nose and throat cancer | H75 |
| Endometrial cancer | X77 |
| Epilepsy | N88 |
| Gastrointestinal ulcer | D85-86 |
| Glaucoma | F93 |
| Heart chronic failure | D72 |
| Hepatoblastoma | D77 |
| Hodgkin/Other lymphomas | B72 |
| Human immunodeficiency virus (HIV) | B90 |
| Hypertension | K86-87 |
| Ischemic heart disease | K74-76 |
| Leukemia | B73 |
| Liver cancer | D77 |
| Lung cancer | R84 |
| Mental retardation | P76 |
| Multiple sclerosis | N86 |
| Obesity | T82 |
| Obstructive chronic pulmonary disease (OCPD) | R95 |
| Osteoarthritis | L89-91 |
| Osteoporosis | L95 |
| Pancreatic cancer | D76 |
| Parkinson | N87 |
| Prostate cancer | Y77 |
| Renal cancer | U75 |
| Renal chronic failure | U80 |
| Retinoblastoma | F74 |
| Schizophrenia | P80 |
| Skin cancer | S77 |
| Soft tissues cancer | L71 |
| Stomach cancer | D74 |
| Stroke | K90-91 |
| Substance abuse | P24 |
| Testicle cancer | Y79 |
| Thyroid cancer | T71 |
| Thyroid disorder | T85-86 |
| Ulcerative colitis | D94 |
| Valvular heart disease | K83 |
| Vasculitis | K99 |
